# The Adsorption of P2X2 Receptors Interacting with IgG Antibodies Revealed by Combined AFM Imaging and Mechanical Simulation

**DOI:** 10.3390/ijms25010336

**Published:** 2023-12-26

**Authors:** Eduardo A. Santander, Graciela Bravo, Yuan Chang-Halabi, Gabriel J. Olguín-Orellana, Pamela A. Naulin, Mario J. Barrera, Felipe A. Montenegro, Nelson P. Barrera

**Affiliations:** 1 Laboratory of Nanophysiology and Structural Biology, Faculty of Biological Sciences, Pontificia Universidad Católica de Chile, Alameda 340, Santiago 8331150, Chile; eas91@cam.ac.uk (E.A.S.); gsbravo@uc.cl (G.B.); gabrielolguinorellana@gmail.com (G.J.O.-O.);; 2 Department of Chemical and Bioprocess Engineering, School of Engineering, Pontificia Universidad Católica de Chile, Santiago 7820436, Chile

**Keywords:** protein adsorption, P2X receptor, protein–protein interaction, membrane protein, AFM imaging, mechanical simulation

## Abstract

The adsorption of proteins onto surfaces significantly impacts biomaterials, medical devices, and biological processes. This study aims to provide insights into the irreversible adsorption process of multiprotein complexes, particularly focusing on the interaction between anti-His6 IgG antibodies and the His6-tagged P2X2 receptor. Traditional approaches to understanding protein adsorption have centered around kinetic and thermodynamic models, often examining individual proteins and surface coverage, typically through Molecular Dynamics (MD) simulations. In this research, we introduce a computational approach employing Autodesk Maya 3D software for the investigation of multiprotein complexes’ adsorption behavior. Utilizing Atomic Force Microscopy (AFM) imaging and Maya 3D-based mechanical simulations, our study yields real-time structural and kinetic observations. Our combined experimental and computational findings reveal that the P2X2 receptor–IgG antibody complex likely undergoes absorption in an ‘extended’ configuration. Whereas the P2X2 receptor is less adsorbed once is complexed to the IgG antibody compared to its individual state, the opposite is observed for the antibody. This insight enhances our understanding of the role of protein–protein interactions in the process of protein adsorption.

## 1. Introduction

Protein adsorption is a crucial event in distinct biological phenomena, like bacteria and cell adhesion [1,2], inflammation response to an implant [3], the blood coagulation cascade [4,5], hemodialysis performance [4], and transmembrane signaling [6], to mention a few. 

Individual protein adsorption is a dynamic bipartite process influenced by various factors. Protein size, charge and spatial orientation, as well as the composition of the surface in contact, play crucial roles [7,8]. Initially, proteins approach the surface in their native state and interact with specific contact residues. The initial orientation of the protein before adsorption is determined by the residues capable of interacting with the surface [7]. Subsequently, favorable protein–surface interactions and an increase in entropy induce a structural reorganization, resulting in the loss of secondary structure and desorption of ions or solvents [7,9]; the “denaturation” of proteins by adsorption on surfaces has been reported since many decades ago [10,11,12]. Consequently, whether adsorbed bioactive proteins such as receptors, antibodies, or enzymes can maintain or change their native state in contact with a solid surface becomes much more relevant, for example, for the sensitivity of diagnostic chips [8,13,14], the activity and stability of the immobilized enzyme [8], biomedical material designing, chromatography retention properties, nanomaterials, and nanotechnology biocompatibility [15,16].

Numerous kinetic and thermodynamic models have been proposed to characterize the process of protein adsorption [17,18,19], primarily focusing on individual proteins and surface coverage. Molecular dynamics (MD) simulations have been employed to investigate protein conformational reorientation upon adsorption, with particular attention given to the atomic interactions between residues and surfaces. For example, Kubiak-Ossowska and Mulheran (2011) studied the adsorption of distinct oligomer states of egg white lysozyme on a charged ionic surface [20] and Javkhlantugs and Bayar (2013) observed three distinct patterns of IgG orientation on a polystyrene surface [21], where both studies elucidated the role of specific amino acids in protein–surface interactions. Furthermore, to enhance the computational efficiency in describing protein–surface descriptions, coarse-grained modeling strategies have been developed, which have demonstrated good agreement with experimental data for individual proteins such as lysozyme, cytochrome C and myoglobin [22]. 

However, the reorientation of protein–protein complexes upon adsorption has been poorly developed due to the lack of combined experimental and analytical strategies. Interestingly, this process can be influenced by the antigenic protein target binding, for example the protein (target)–protein (antibody) complex would become key for a model to control or predict the adsorption efficiency, rather than considering independent protein components. Furthermore, how this protein–protein complex is oriented towards the adsorption mechanism might provide important information to conserve antigen accessibility or bioactivity [9]. Via atomic force microscopy (AFM) imaging and Quartz Crystal Microbalance with Dissipation (QCM-D), it was found that individual IgG antibody adsorption initially occurs predominantly in a flat-on orientation (three structural fragments lying flat on the hydrophilic [23] or hydrophobic surfaces [24]). Later, when the surface becomes crowded, a mixture of other vertical orientations is present [24]. However, it remains unclear how the preferred orientation of adsorbed IgG triggers conformational reorientations upon surface contact, which, as indicated above, can be of much relevance once it is complexed to the target protein. 

Previously, a novel computational approach was performed using the Autodesk Maya 3D software to characterize the three-dimensional interactions between bacteria and nano-structured surfaces [25]. The capabilities of Maya, specifically its Dynamics and nDynamics modules, allowed for the construction and visualization of plausible scenarios depicting bacterial interactions with these surfaces. In this approach, topographic parameters obtained from AFM imaging were imported into Maya. Experimental data, including the visualization of bacterial cells interacting with titanium surfaces, were used to inform the construction of these scenarios [25]. This represents one of the pioneering attempts to integrate structural AFM data into the physical embedded parameters of well-known 3D visualization software. In addition, this 3D software has also been used as a complementary method to physically model a variety of tissues including vastus medialis muscle [26], levator veli palatini muscle [27], and extra- or intramuscular innervation throughout the muscle volume of the extensor carpi radialis longus and brevis [28], which allowed the authors to propose a functional reconstruction of muscle movement patterns. Taken together, this evidence strongly supports the idea of applying this 3D software to simulate dynamical processes occurring in biological contexts where macromolecules such as proteins interact with each other upon adsorption.

Herein, our work investigated the irreversible adsorption process of the multiprotein complex formed by an anti-His6 IgG antibody bound to the His6-tagged P2X2 receptor. This study utilized a combination of AFM imaging and a computational approach based on Maya for mechanical simulations at a second-scale resolution. By employing a single-molecule level approach, we were able to determine the real-time structural and kinetic effect of protein adsorption, offering a more detailed understanding of the molecular mechanisms involved and the influence of protein–protein interaction on protein adsorption onto surfaces.

## 2. Results

**Figure 1:**
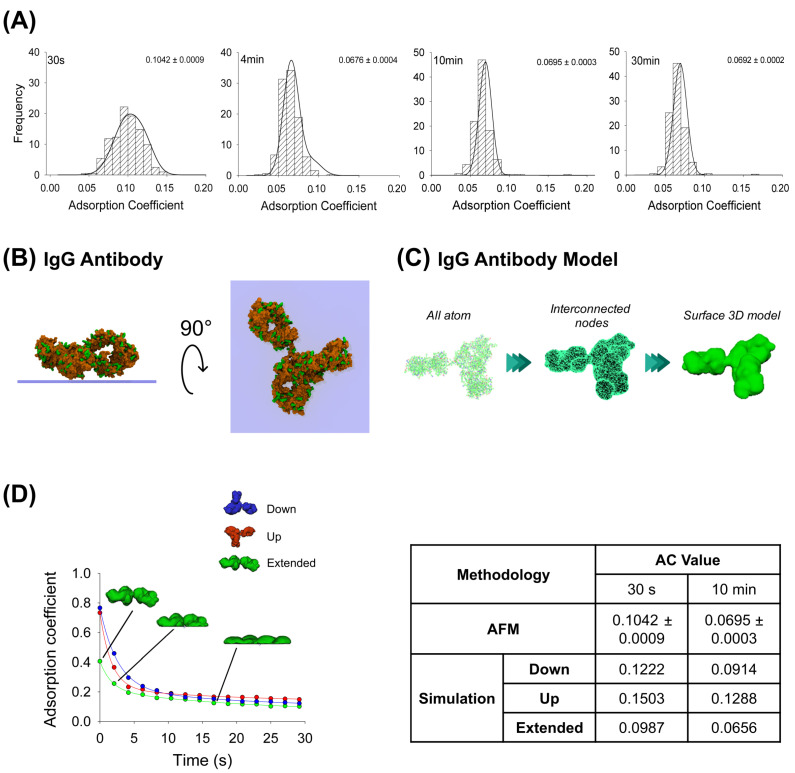
Adsorption analysis of anti-His6 IgG antibody by mechanical simulation and AFM. (**A**) Frequency distribution of adsorption coefficient (AC) of anti-His6 antibody adsorbed onto mica muscovite in different incubation times: 30 s, 4 min, 10 min, and 30 min. The curves indicate fitted Gaussian functions and the numbers indicate the peak ± SEM. (**B**) Top and lateral views of IgG antibody showing surface basic residues favorable for the adsorption (colored green). (**C**) Structural representation models of the IgG antibody. Three different structures were generated. All-atom representation considers all the residues and was taken to make the final simulation model; this was obtained from the PDB database (PDB ID: 1HZH). Interconnected nodes structure represents the set of nodes that are connected by elastic springs forming deformable solid bodies. The surface 3D model was used for simulation in Maya and represents a mesh created from the all-atom structure. (**D**) IgG antibody adsorption kinetics in different orientations (down, up and extended) by mechanical simulations up to 30 s. Structures of antibody extended orientation are shown through the adsorption process. The table shows the AC comparison considering both methodologies and orientations in simulation at two different times, 30 s and 10 min. The values for 10 min in the simulation were extrapolated using a corresponding fitting equation.

AFM images were obtained at the single-molecule level for the IgG antibody’s, the P2X2 receptor’s and the multi-protein complexes’ adsorption. After incubating the IgG anti-His6 antibodies onto mica for different periods, the particles were imaged using AFM and then the adsorption coefficient (AC) was calculated for each particle adsorbed (more details in Methods, Section 4.5). As expected, histograms depicted in Figure 1A indicate a range of ACs for each incubation time. Statistically, these results indicate that the particles at 30 s incubation (AC peak 0.1042 ± 0.0009, n = 203) were significantly different (*p* < 0.05) than incubations at longer times. Also, samples at 4 min (AC peak 0.0676 ± 0.0004, n = 313) were different to longer adsorption times of 10 min (AC = 0.0695 ± 0.0003, n = 296) and 30 min (AC peak 0.0692 ± 0.0002, n = 329). However, no significant differences in AC values were observed between 10 and 30 min incubation times (*p* > 0.05). It has been shown that a high adsorption degree is correlated to a decrease in particle height and an increase in its maximum diameter [29]. Moreover, it is known that protein adsorption is time-dependent when in contact with the surface, which experimentally was observed to reach a lower adsorption coefficient peak when proteins were more adsorbed. AFM imaging demonstrated that the adsorption degree did not change after 4 min of incubation, which could be because the protein reached a stable conformation over the mica, preventing further adsorption.

Additionally, MD simulations were applied to elucidate possible configurations of the antibody adsorbed on the mica substrate. Both favorable (positively charged residues) and unfavorable (negatively charged residues) interactions between the protein residues and the negatively charged mica surface were taken into account to set up the initial orientation of the protein (Figure 1B), pursuing the facilitation of its adsorption (more details in Methods, Section 4.2), which revealed that the highest affinity score is associated with the extended configuration of the IgG antibody (Table 1). Employing this computational technique, we generated structural inputs for the mechanical simulations in Maya, where we aimed to gain a deeper understanding of the process over extended durations and the potential configurations that would arise; we conducted a surface three-dimensional description model simulation to investigate the dynamics of the adsorption. The method was possible to carry out considering Maya’s nDynamic feature, which conserves the internal volume of the object while exerting low stretching resistance and damping [25]. Figure 1C illustrates the IgG all-atom structure as well as generating interconnected nodes at the maximum mesh resolution to reproduce the IgG 3D surface model, which was used for the simulations in Maya.

This computational dynamic method allowed us to generate self-collision events between the protein and the surface by applying external vertical dragging forces to study the adsorption kinetics and to understand how the initial IgG antibody orientation influences the protein adsorption process using three different orientations (up, down and extended). The IgG antibody in its extended orientation adsorbs more than the other orientations over the 30 s simulation, while the up-orientation is more adsorbed than the down-orientation in the first 9 s; this behavior is reversed for the rest of the simulation (Figure 1D). A summary for either AFM imaging or fitted mechanical simulation (parameters summarized in Table 2) at 30 s and 10 min (extrapolated value) is shown in Figure 1D. It can be seen that the IgG antibody-extended orientation AC values are quite similar to the values obtained by AFM imaging at 30 s (0.0987 and 0.1042 ± 0.0009, respectively) and 10 min (0.0656 and 0.0695 ± 0.0003, respectively). This could suggest that the majority of the particles might adsorb in extended orientation. This dynamical adsorption behavior is also observed if the IgG 3D model is built from a lower number of interconnected nodes that reduces the mesh resolution of the protein, creating sphere-like shapes of the Fab and Fc domains (Supplementary Figure S1). As the resulting number of interactions between the protein and surface are considerably reduced, the AC values reflect a higher adsorption rate (0.0510 for the extended orientation and 0.0838 for the up-orientation at 28 s simulation) due to less time required for a complete structure rearrangement after collision. Taken together, these results support the application of a dynamical approach based on collisions where the modelled mesh protein should resemble all-atom structures to characterize the adsorption process.

**Figure 2:**
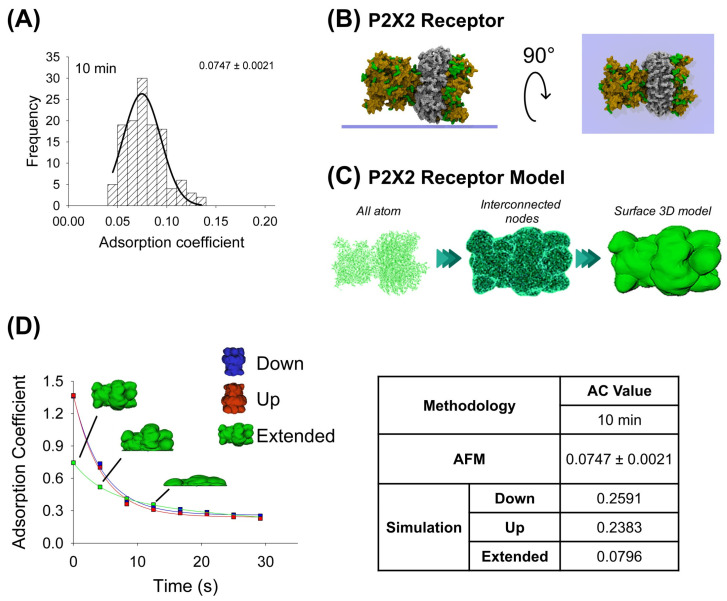
Adsorption analysis of P2X2 receptor by mechanical simulation and AFM. (**A**) Frequency distribution of adsorption coefficient of P2X2 receptor adsorbed onto mica muscovite at 10 min of incubation. The curve indicates a fitted Gaussian function and the number indicates the peak ± SEM. (**B**) Top and lateral views of P2X2 receptor showing its surface basic residues favorable for the adsorption (colored green). (**C**) Structural representation models of the receptor. Three different structures were generated. All-atom representation considers all the residues and was taken to make the final simulation model, this is obtained by homology modelling (more details in Methods Section 4.1 and Section 4.3). Interconnected nodes structure represents the set of nodes that are connected by elastic springs forming deformable solid bodies. The surface 3D model was used for the simulation in Maya. It represents a Mesh created from structure all-atom. (**D**) P2X2 receptor adsorption kinetics in different orientations (down, up and extended) by mechanical simulations up to 30 s. Structures of the receptor in extended orientation are shown through the adsorption process. The table shows the AC comparison considering both methodologies and orientations at 10 min. The values for 10 min in the simulation were extrapolated using the corresponding fitting equation.

In the same way, the purified P2X2 receptor preparation was adsorbed for 10 min onto a mica support, dried by nitrogen gas, and imaged using AFM when air-exposed (Figure 2A). Then, by the favorable and non-favorable interactions calculation, it was observed that the highest affinity score is associated with the extended configuration for the P2X2 receptor (Table 1). The P2X2 all-atom structure was taken to make the interconnected nodes and surface 3D models used for mechanical simulations. Similarly, the P2X2 extended orientation is also the most adsorbed orientation (0.0796), once extrapolated to 10 min, compared to the down- and up-orientations (0.2591 and 0.2383, respectively). Furthermore, this extended orientation is more similar to that obtained from AFM imaging (AC peak 0.0747 ± 0.0021, n = 90) (Figure 2A,D). Note that, in contrast to antibody adsorption, P2X2 down and up orientations have a similar adsorption kinetics behavior, which could be explained due to its structure being more compact than the antibody (Table 2).

**Figure 3:**
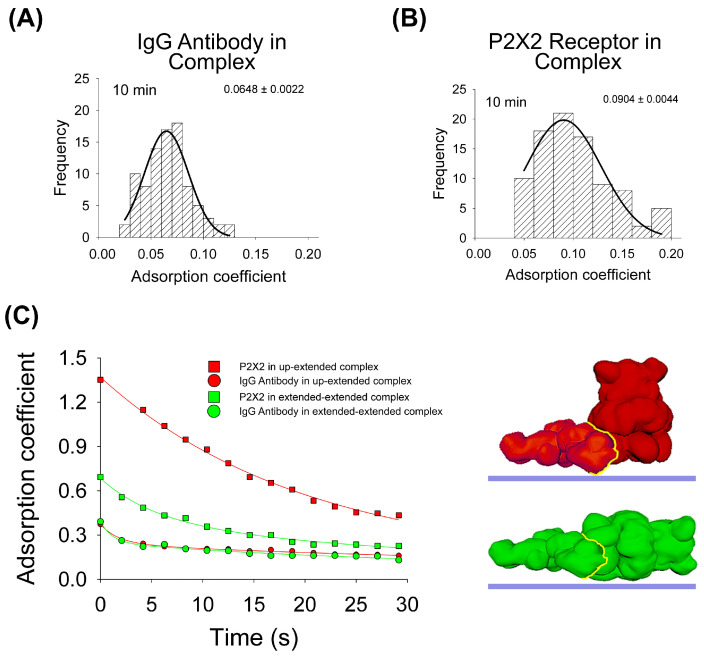
Adsorption analysis of antibody–receptor complex by mechanical simulation and AFM. Frequency distribution of adsorption coefficient of anti-His6 antibody (**A**) and P2X2 receptor (**B**) within the complex adsorbed onto mica at 10min of incubation and imaged by AFM. The curves indicate fitted Gaussian functions and the numbers show the AC peak ± SEM. (**C**) Structural 3D surface models of the antibody–receptor complex, partially and totally extended, used for mechanical simulations. Adsorption kinetics of the two complexes are shown up to 30 s in partially and completely extended orientations. The fitted curves indicate five-parameter exponential double decay functions. Red and green structures represent both orientations of P2X2–IgG complexes. The yellow line indicates the binding interface.

To test the influence of the protein–protein interaction in the adsorption process, the same experimental and computational approaches were developed. Regarding AFM imaging, purified P2X2 receptors complexed with anti-His6 IgG antibodies were incubated onto mica for 10 min, dried and then imaged by AFM. As for the antibodies, the data indicates that the antibody adsorption of the majority of the particles is larger (*p* < 0.05) after 10 min when the antibody forms part of the complex (AC peak 0.0648 ± 0.0022, n = 89, Figure 3A), compared to its adsorption alone (AC peak 0.0695 ± 0.0003, n = 296, Figure 1A). On the contrary, P2X2 receptors are more adsorbed (*p* < 0.05) when alone (AC peak 0.0747 ± 0.0021, n = 90, Figure 2A) than joined to the IgG antibody (AC peak 0.0904 ± 0.0044, n = 92, Figure 3B). In addition, the AC value distribution is wider for proteins forming complexes than being adsorbed alone which suggests a larger heterogeneity in the adsorption process.

Then, for the adsorption simulation studies of the IgG–P2X2 protein complex, two different surface models were made, as shown in Figure 3C. In both cases, the IgG antibody adopted an extended orientation, which was bound to either P2X2 in its extended orientation or P2X2 in its up-orientation. The IgG–P2X2 extended–extended complex presented the larger affinity score (Table 1), with the P2X2 receptor showing a higher AC value after forming the complex (0.0991) compared to when adsorbed alone (0.0796) at 10 min. Whereas the IgG antibody behaves in the opposite way, when it is adsorbed alone (0.0656), it has a higher AC value than when forming the complex (0.0618). Surprisingly, our simulations revealed a distinct pattern for the IgG–P2X2 extended–up-orientation complex. In this scenario, the P2X2 receptor adsorbed more when forming the complex (0.1112) than when alone (0.2383), while the opposite was shown for the IgG antibody (0.1041 in the complex and 0.0656 alone) (Figure 3C). When we compared these simulation results with our AFM data collected at 10 min, the IgG–P2X2 extended–extended complex displayed an AC behavior more closely aligned with the experimental data. Taken together, these results suggest that most of the complexes are adsorbed in an extended–extended orientation structure, following a greater interaction of favorable residues expected to occur once in contact with the mica.

## 3. Discussion

Mechanical simulations have enabled the acquisition of protein adsorption kinetics at the individual molecule level, exhibiting time courses comparable to experimental results. This applies even to multiprotein complexes, which cannot be achieved through all-atom molecular dynamics due to the substantial computational resources needed [22,30]. Recent studies investigating protein–protein interactions through coarse-grained simulations have primarily operated with a time range of nanoseconds to microseconds [31,32,33]. However, our conducted simulations have significantly exceeded these timeframes, enabling us to capture extensive long-term dynamics within the systems. We chose Maya3D with the mMaya v1.3 plugin, considering how it offers the best balance between structural complexity and simulation time required for our study. This expanded time coverage has enabled a thorough comparison between our computational findings and experimental observations obtained using AFM. Notably, our utilization of a physical spring/mass approach conserving internal particle volume and applying constant dragging forces over an all-atom-derived protein 3D surface model has effectively replicated the intricate conformational alterations observed in the proteins, such as the pivotal hinge motion, and within the protein–protein complex, in order to propose preferred orientations of adsorbed proteins.

Several reports propose that the adsorption of monoclonal IgG presents an extended or “flat-on” orientation at low IgG surface density, with their Fc and Fab fragments lying flat on the surface [23,34,35,36]. In our study, the examination of the IgG antibody adsorption process, revealed by experimental and computational outcomes, indicated a consistent observation of antibody adsorption occurring preferentially in an extended configuration over the mica substrate. This orientation is suggested to be influenced by surface chemistry, the presence of other proteins, salinity, and pH conditions [23,37,38,39]. The relationship between IgG orientation on solid surfaces and antigen binding capacity is of interest [23,40,41]; for example, Xu H et al. (2006) [23] revealed an increased steric hindrance with a higher antibody packing density, affecting hCG binding. Additionally, hCG binding is pH-dependent, with maximum binding around the isoelectric pH of the antibody, and is influenced by steric hindrance and electrostatic interactions under increased ionic strength. However, it is unclear how the contributions of the Fab and Fc regions change during adsorption with antigen binding. We have shown that IgG antibody exhibits a higher adsorption when forming a complex compared to the antibody alone. It is reasonable, then, to assume that Fc and Fab domains are involved in the adsorption of the antibody, either alone or in a complex with a P2X2 receptor. However, Fab domain movement is restricted by the additional binding to the His6 tag antigen in the receptor during adsorption, which may induce conformational and physicochemical changes in the antibody [34,42], affecting its surface properties and speeding up adsorption behavior.

As observed in adsorption simulations, the IgG antibody exhibits a faster adsorption compared to the receptor while forming the complex, even when their individual affinity values are equal. As discussed throughout this article, protein adsorption is a complex phenomenon; however, it has been shown that electrostatic interactions contribute substantially to IgG1 adsorption to siliconized glass [43], and, by all-atom MD simulations, it has been observed that major driving forces for the adsorption of IgG onto the polystyrene surface come from serine, aspartic acid, and glutamic acid residues [21]. Perhaps, under conditions of low-IgG coverage and medium salinity, it is possible to predict the overall predominant orientation of the antibody–antigen complex with respect to the planar surface only by the favorable and unfavorable residues of different conformations, knowing the protonation state of the residues of each protein and electrical properties of a surface. We determined that the fully extended complex has a higher affinity index (Table 1), and this was the initial orientation that best matches what was observed experimentally. However, the kinetic and adsorption process differences require other descriptive aspects, such as dipole moments in IgG adsorption on solid surfaces [44], or could be due to some more flexible domains in the antibody, whereas the micelle structure would reduce the degrees of freedom of the P2X2 receptor.

The protein–protein orientation during adsorption seems to have relevance for the complexes experimentally adsorbed, since the kinetics of the IgG’s ‘extended orientation’ complexed to P2X2 receptor’s ‘up-orientation’ may represent a minority in the adsorption process. This lines up with the study of Hu et al. (2019), who proposed that the physical adsorption process for biotinylated antibody-specific Immunoglobulin E (IgE) immune complexes must occur in a specific manner. For example, if an IgE antibody is present in a random orientation, only a small fraction of the adsorbed antibodies can capture biotinylated antibodies and retain them [45].

It has been revealed that a considerable number of proteins exhibit an initial loose binding to the surface, gradually enhancing their affinity through subsequent structural modifications [7], which in turn significantly influences the kinetics of protein adsorption. Initially, a newly formed protein layer cannot resist elution. However, after a certain time interval, spanning from a few minutes to several hours, proteins may become firmly and irreversibly attached to the surface [46], inducing, in consequence, changes in AC values that could influence the biological functionality of the protein [47,48]. Based on our findings, it is plausible to propose that the presence of IgG antibodies ensures the capability to modulate the degree of adsorption, thereby influencing the conformational state of the target protein. By strategically harnessing the empirical utilization of antibody presence, it becomes possible to enhance sensitivity and overall performance in these applications. Moreover, our experimental and computational results indicate that a significant portion of the IgG antibody, receptor, and their complex are absorbed in an “extended” orientation; this observation holds significant implications, particularly in the ongoing development of detection kits and biosensors, highlighting the importance of considering these adsorption characteristics [49,50,51].

## 4. Materials and Methods

### 4.1. Homology Modelling of the P2X2 Receptor

The crystallized zebra fish P2X4 receptor in the apo state (PDB ID: 4DW0) was used as a template for modelling the rat P2X2 receptor (UNIPROT ID: P49653) using MODELLER 9v13 [52]. The final structure was selected with the lowest DOPE score. It was further assessed by RAMPAGE [53] for the Ramachandran plot of the distribution of those allowed residues and outlier regions. The energetic quality of 3D models was verified by ProSA (Protein Structure Analysis) [54]. N- and C-terminuses were modelled using MODELLER 9v13 and their secondary structure was included using I-TASSER server ‘https://zhanggroup.org/I-TASSER/’ (accessed on 15 October 2015).

### 4.2. The Determination of the Initial Favorable Orientation of the Proteins on the Surface

In the context of protein adsorption, in order to establish the initial orientation of the proteins on the surface, a script named “proteinInitialPosition.tcl” was developed. This script aims to identify the orientation that facilitates the interactions between the protein (or the multiprotein complex) with the adsorbent, employing a series of parameters specified by the user. The parameters that the “proteinInitialPosition.tcl” script received as input were two coordinate files in .pdb format containing the protein or complex structure and the surface; an angle indicating the degrees by which the input structure would be rotated in each of the Cartesian plane axes to explore its possible initial orientations concerning the surface (15°); a cut-off distance from an implicit mica surface to the inside of the input structure for counting the favorable and unfavorable residues from a chemical perspective, mimicking the electrostatic and van der Waals interactions (12 Å); the number of basic residues that have been defined as favorable (LYS, ARG, HIS) and unfavorable acid residues (GLU, ASP) for interacting with the substrate; and the distance that the protein would be from the surface after determining the initial orientation (2 Å) for the simulations in Maya 3D.

The favorable and unfavorable residues were chosen taking into account the negative charge surface of the basal layer of the mica muscovite, which was the adsorbent. Due to the definition of these residues before the execution of the script, it was not necessary to use the mica to calculate the affinity of the interactions but it was necessary to calculate the final distance between the protein and the surface.

Given these parameters, proteinInitialPosition.tcl applied sequential rotations to the input structure. The process initiated with rotations around the x-axis, followed by the first rotation around the y-axis when finished. Then, from the starting point on the x-axis, the process started again. This iterative process persisted for a constant orientation along the z-axis and only after the completion of all the y-axis rotations, the script triggered a rotation around z, replicating the procedure once more. Since an angle of 15° was selected in this procedure, 360°/15° = 24 rotations in each axis of the Cartesian plane were applied. Then, a total of 24 × 24 × 24 = 13,824 orientations were sampled for each input structure and the affinity value was calculated as the difference between the favorable and unfavorable residues.

After determining the initial orientation for each one of the input protein structures, NAMD 2.9 [55] with the INTERFACE v1.5 force field [56] was used to carry out 10 ns MD simulations in an NPT ensemble (298 K; 1 atm). Explicit water molecules (TIP3P models) and a three-layer mica muscovite surface were considered, and a harmonic restriction of 1 kcal/mol was applied in every atom to reproduce the rigidity of the material. Additionally, it was allowed to vary their dimensions along the z-axis, while a constant relationship between the x- and y-cell vectors was imposed to optimally represent the anisotropy of the surface. Then, the resulting atomic configurations were used as the input for Maya 3D’s mechanical simulations.

### 4.3. The Formation of the P2X2 Receptor–Micelle Structure

The receptor–micelle model structure was performed by PACKMOL [57] using CHAPS as a zwitterionic detergent [58]. A number of 110 detergent molecules (approx. 10 times CMC of CHAPS) was used to fully cover the transmembrane region of the P2X2 receptor.

### 4.4. The Mechanical Simulation of the Adsorbed Individual Proteins and Complexes via Autodesk Maya 3D

All-protein models, antiVIH-1 b12 IgG antibody (PDB ID: 1HZH), P2X2 receptor and P2X2 receptor–IgG complex were imported into Autodesk Maya 3D v2011 using the mMaya v1.3 (Molecular Maya Toolkit) plugin, which allows for integration onto simulations within Maya. 

Simulations were conducted using Maya’s nDynamic feature. All-atom structures sourced from the PDB database were transformed into interconnected nodes, which were then used to form a 3D surface model. This 3D-surface structure served as the basis for simulations in Maya, representing a polygonal mesh created from the all-atom structures. Thus, during adsorption simulations, we only evaluated the physical aspect (collision and deformation), excluding the chemistry of the residues which interacted between the protein and the surface [25], except for the initial orientations of the proteins prior to adsorption.

The adsorption process of the particles was measured as a passive collision (no additional force used) of the non-rigid particle onto a surface, and involving a particle deformation upon adsorption (noting that all adsorbed particles underwent structural changes). Thus, internal volume of the object and dragging force applied were conserved during simulation.

Spring/mass model was used in simulations; these are performed considering the proteins as set-of-nodes which are interconnected by elastic springs forming deformable solid bodies which follow Hooke’s law. The vertical dragging force is applied under these nodes, allowing dynamic deformation of the particles when they adsorb onto the surface, while maintaining the particle volume during their spreading out onto the surface. AC values were calculated every 2–4 s during simulation (24 frames/s), considering the height and radius at the base of the particle. For all the studied models, the simulation time was set to a maximum of 30 s.

### 4.5. AFM Imaging of the IgG Antibody, P2X2 Receptor and Receptor–Antibody Complex

AFM imaging was performed with a multimode atomic force microscope (Digital Instruments, Santa Barbara, CA, USA) in air-tapping mode, using silicon cantilevers with spring constant of 40 N/m (Mikromasch, Sofia, Bulgaria), amplitude setpoint average 1.4 V, and drive frequency of ∼300 kHz. The experimental adsorption analysis of IgG, receptors, and complexes was determined from particle dimensions based on AFM images. Mouse monoclonal anti-His6 IgG antibody (Research Diagnostics Inc., Baileys Harbor, WI, USA) was adsorbed for 30 s, 4 min, 10 min, and 30 min over mica, then washed with milliQ water and dried with nitrogen gas prior to AFM imaging. AFM images of the P2X receptor incubated alone or with anti-His6 IgG, adsorbed 10 min over mica, were obtained previously [59]. The focus of that study was to obtain the subunit stoichiometry of the receptor, whereas, herein, its raw data was analyzed differently as follows to calculate topographical measures of the protein adsorption process. After adsorption of the proteins onto the mica, the particles adopted the shape of a spherical cap. The adsorption coefficient (AC) was calculated using the following equation:

AC = *h*/2*R_C_*, (1)

where *h* is the particle height and *R_C_* is the corrected radius at mica contact, derived from measured radius at half-height to prevent the effect of tip convolution in the scanning [60], after averaging multiple cross-sections of the same particle.

### 4.6. Statistical Analysis

One-way ANOVA followed by a post hoc Tukey test was used to determine if the AC values of IgG antibody groups after AFM imaging (4 s, 30 s, 10 min, 30 min) were statistically different (*p* < 0.05). In addition, t-test was used to statistically compare (*p* < 0.05) AC values of IgG antibody or P2X2 receptors between their individual or complex-forming states after AFM imaging.

## 5. Conclusions

This study has illustrated the potential of Autodesk Maya 3D in generating high-fidelity simulations of the interactions between single proteins or multi-protein complexes and substrate surfaces to evaluate AFM data. Furthermore, it has contributed to a deeper understanding of the adsorption kinetics and protein orientation during the adsorption process.

## Figures and Tables

**Table 1:** Antibody, receptor and multiprotein complexes affinity analysis. The table shows the affinity of the proteins with the mica surface expressed as the difference between the favorable (basic) and unfavorable (acidic) residues for the antibody, receptor and both multiprotein complexes analyzed in their most favorable orientation for the adsorption (complete and partially extended).

Model	Favorable Residues	Unfavorable Residues	Affinity
IgG Antibody	14	7	7
P2X2 Receptor	10	3	7
Complex complete extended (IgG extended/P2X2 extended)	24	10	14
Complex partially extended (IgG extended/P2X2 Up)	14	9	5

**Table 2:** Non-lineal regression parameters’ values for the antibody, P2X2 receptor and the multiprotein complex used to fit the AC values obtained by the mechanical simulation.

Simulation			Regression Parameters					
Protein	Condition	Orientation	y0	a	b	c	d	R sqr
Antibody	Individual	Down	0.0914	0.5378	0.3951	0.1381	0.0500	0.9993
		Up	0.1288	0.5086	0.6070	0.0967	0.0500	0.9989
		Extended	0.0656	0.1937	0.6112	0.1485	0.0500	0.9971
	Complex	IgG Extended/P2X2 Up	0.1041	0.1283	0.5769	0.1406	0.0300	0.9917
		IgG Extended/P2X2 Extended	0.0618	0.1426	0.8480	0.1867	0.0300	0.9810
Receptor	Individual	Down	0.2591	1.1087	0.2213	5.498e^−13^	0.0270	0.9948
		Up	0.2383	1.1296	0.2312	0.0050	0.0270	0.9967
		Extended	0.0796	0.3111	0.2208	0.3538	0.0270	0.9998
	Complex	P2X2 Up/IgG Extended	0.1112	1.2584	0.0501	7.442e^−13^	0.0350	0.9963
		P2X2 Extended/IgG Extended	0.0991	0.2684	0.1939	0.3168	0.0350	0.9920

The non-linear fit used corresponds to a five-parameter exponential double decay whose equation is y = y0 + ae^−bx^ + ce^−dx^. All simulation data are significantly fitted to the regression (*p* < 0.05).

## Data Availability

The data presented are contained within the article and the proteinInitialPosition.tcl script is publicly archived in ‘https://github.com/gabrielolguinorellana/proteinInitialPosition’ (accessed on 18 November 2023).

## Supplementary Materials

The following supporting information can be downloaded at: https://www.mdpi.com/article/10.3390/ijms25010336/s1.
