# Supplementary material for: The Adsorption of P2X2 Receptors Interacting with IgG Antibodies Revealed by Combined AFM Imaging and Mechanical Simulation
Source: Int J Mol Sci. 2023 Dec 26;25(1):336. doi: 10.3390/ijms25010336 (PMC10778698; doi:10.3390/ijms25010336)
Supplement: Supplementary file 1 [file ijms-25-00336-s001.zip › ijms-2718985-supplementary.pdf]

Supplementary Material

Article

The Adsorption of P2X2 Receptors Interacting with IgG Antibodies Revealed by Combined AFM Imaging and Mechanical Simulation

Eduardo A. Santander [1,+], Graciela Bravo [1,2,+], Yuan Chang-Halabi [1], Gabriel J. Olguín-Orellana [1], Pamela A. Naulin [1], Mario J. Barrera [1], Felipe A. Montenegro [1], and Nelson P. Barrera [1,*]

[1] Laboratory of Nanophysiology and Structural Biology, Faculty of Biological Sciences, Pontificia Universidad Católica de Chile, Alameda 340, Santiago 8331150, Chile
[2] Department of Chemical and Bioprocess Engineering, School of Engineering, Pontificia Universidad Católica de Chile, Santiago 7820436, Chile
[+] These authors contributed equally to this work.
[*] Correspondence: nbarrera@bio.puc.cl

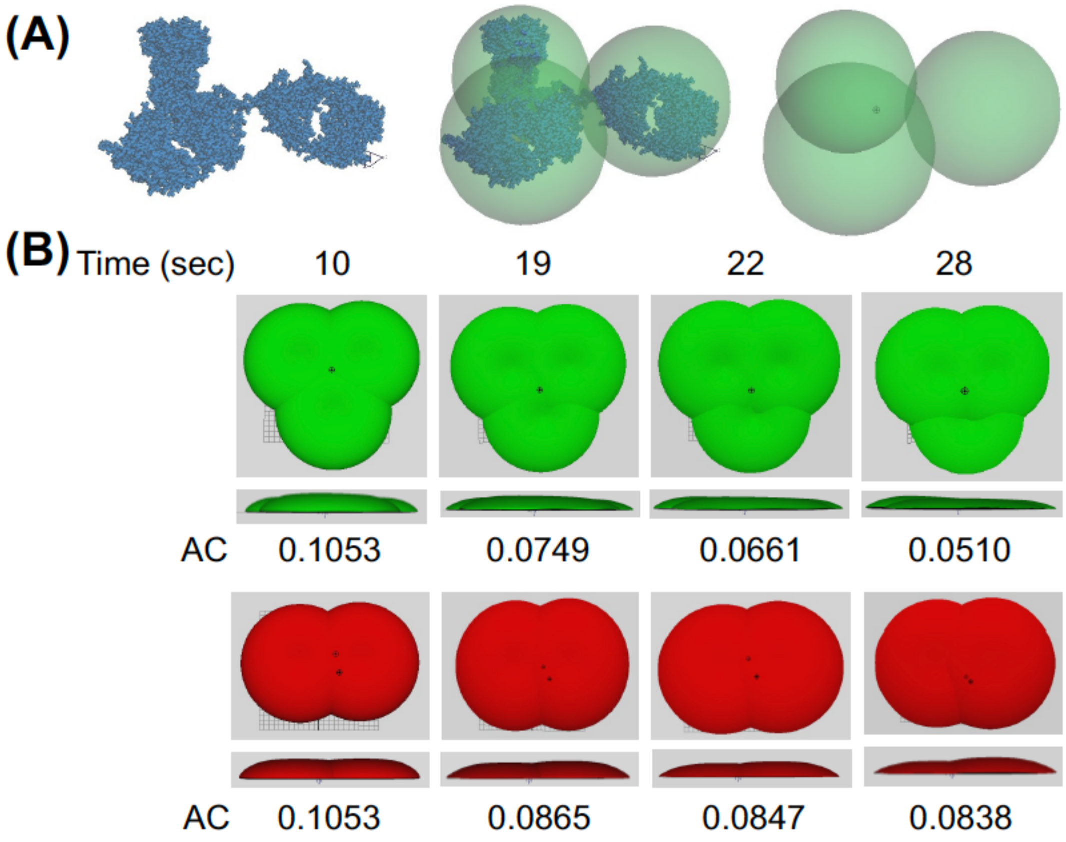

Supplementary Figure S1. Adsorption analysis of anti-His6 IgG antibody by mechanical simulation under reduced number of interconnected nodes. **(A)** All-atom structural representation model of the IgG antibody (PDB ID: 1HZH) in the extended orientation, followed by the interconnected nodes model and the 3D sphere-like shape model used for simulation., **(B)** Snapshots of the adsorption process of IgG antibody model in extended orientation (top) and up-orientation (bottom). Corresponding time and AC values during adsorption simulations are indicated.
